# Broad Resistance to ACCase Inhibiting Herbicides in a Ryegrass Population Is Due Only to a Cysteine to Arginine Mutation in the Target Enzyme

**DOI:** 10.1371/journal.pone.0039759

**Published:** 2012-06-29

**Authors:** Shiv Shankhar Kaundun, Sarah-Jane Hutchings, Richard Paul Dale, Eddie McIndoe

**Affiliations:** Syngenta, Jealott's Hill International Research Centre, Biological Sciences, Bracknell, United Kingdom; University of Umeå, Sweden

## Abstract

**Background:**

The design of sustainable weed management strategies requires a good understanding of the mechanisms by which weeds evolve resistance to herbicides. Here we have conducted a study on the mechanism of resistance to ACCase inhibiting herbicides in a *Lolium multiflorum* population (RG3) from the UK.

**Methodology/Principal Findings:**

Analysis of plant phenotypes and genotypes showed that all the RG3 plants (72%) that contained the cysteine to arginine mutation at ACCase codon position 2088 were resistant to ACCase inhibiting herbicides. Whole plant dose response tests on predetermined wild and mutant 2088 genotypes from RG3 and a standard sensitive population indicated that the C2088R mutation is the only factor conferring resistance to all ten ACCase herbicides tested. The associated resistance indices ranged from 13 for clethodim to over 358 for diclofop-methyl. Clethodim, the most potent herbicide was significantly affected even when applied on small mutant plants at the peri-emergence and one leaf stages.

**Conclusion/Significance:**

This study establishes the clear and unambiguous importance of the C2088R target site mutation in conferring broad resistance to ten commonly used ACCase inhibiting herbicides. It also demonstrates that low levels “creeping”, multigenic, non target site resistance, is not always selected before single gene target site resistance appears in grass weed populations subjected to herbicide selection pressure.

## Introduction

Modern herbicides are very effective tools for controlling weeds in agricultural production. Their extensive use over time, however, has resulted in evolution of weed resistance to key herbicide modes of action [1]. This is often the case when herbicides are employed as the sole method of weed control combined with little to no diversity in agronomic practices [2]. Weeds prone to evolve resistance are generally highly prolific, genetically diverse, out crossing species [3]. This is exemplified by ryegrasses which have evolved resistance to practically all herbicide modes of action that are active against sensitive populations of this species [4], [5]. Particularly affected are highly effective single site herbicides such as inhibitors of acetolactate synthase (ALS) [6], [7], [8] and acetyl CoA carboxylase (ACCase) [9], [10], [11].

The first case of ryegrass resistance to an ACCase herbicide was reported in 1982 in a wheat field in Australia [12]. Over the last 30 years many more ryegrass populations have evolved resistance to ACCase herbicides across five continents, including France, UK, Germany, Spain, Italy, Canada, USA, Chile, Brazil South Africa, Tunisia, Greece, Israel, Iran, Saudi Arabia and Australia [5]. The situation is particularly alarming in Australia with confirmed resistance in over 70% of ryegrass populations sampled randomly in the wheat belt. A majority of these populations are also resistant to other herbicide modes of action thus complicating weed management strategies [13].

Herbicides targeting acetyl CoA carboxylase inhibit the synthesis of *de novo* fatty acids which are essential components in cell membranes and secondary plant metabolites [14]. Three classes of ACCase herbicides have been developed, namely cyclohexanediones (DIMs), aryloxyphenoxypropionate (FOPs) and phenylpyraxoline (DEN) [15]. All three herbicide classes are active on the chloroplastic form of most grass ACCase with little to no activity on broadleaved species [16]. The difference in herbicide activity results from the dissimilar forms of chloroplastic ACCase in grass and broadleaves weeds. In dicotyledonous plants, plastidic ACCase is multisubunit consisting of biotin carboxylase (BC), biotin-carboxyl carrier protein (BCCP) and carboxyl-transferase (CT). In contrast chloroplastic ACCase is multidomain with a large functional polypeptide comprising of BC, BCCP and CT in most grass weeds [17].

ACCase inhibiting herbicides bind to the target enzyme in a near competitive manner with respect to the substrate acetyl-CoA. Early double inhibition studies showed that the FOPs and DIMs are mutually exclusive indicating that they share a common binding site [18]. Recently, crystal structures of the CT domain in complex with haloxyfop and tepraloxydim revealed that the two herbicides are bound in the active site of the CT domain, particularly at the interface of the dimer [19], [20]. The DIM and FOP herbicides probed distinct regions of the dimer interface sharing only two main anchoring points on the ACCase enzyme. Pinoxaden and tepraloxydim were found to bind in a very similar location on the ACCase target in spite of their very different chemical structures [21].

Resistance to ACCase herbicides can be due to enhanced metabolic degradation of the toxophore or insensitivity of the target enzyme. Metabolism is complex and involves several genes that are gradually selected and combined over several generations [22], [23], [24], [25]. Target site resistance results from a single amino acid change in the ACCase enzyme. Mutations at seven ACCase codons have been reported to date and include positions 1781, 1999, 2027, 2041, 2078, 2088 and 2096 (*Alopecurus myosuroides* equivalent) [26], [27]. Resistance conferred by target site mutations can be broad or specific and strong or weak, in part, reflecting the different binding modes of the three classes of ACCase herbicides [26]. The cross resistance patterns between current commercial ACCase herbicides have been established for some but not all these resistance mutations and weed species. In many cases, the contribution of additional underlying non target site resistance mechanisms have not been taken into consideration. Investigating the mechanisms of resistance and exploiting the differences in activities between ACCase inhibiting herbicides is of importance given the limited options for controlling grass weeds in cereal and broad leaved crops [28]. Here we have conducted a thorough biological and molecular study on the mechanism of ACCase resistance in a *Lolium multiflorum* population (RG3) from the UK. Ten most commonly used ACCase herbicides were investigated for their efficacies against the suspected resistant ryegrass population. Additionally the influence of plant growth stages was investigated on wild and mutant RG3 genotypes for the most potent ACCase herbicide, and a DNA based marker method was developed for large scale identification of the target site resistant mutation identified in this study.

## Results

### Resistance confirmation test

The standard sensitive population STD1 was killed with field rates of clodinafop-propargyl, cycloxydim and pinoxaden. In contrast the three FOP, DIM and DEN herbicides provided only partial and similar levels of control for population RG3. On average the percentage biomass reduction was around 30% relative to the untreated control for all three herbicides. There were either dead or very healthy plants in the treated pots indicating that RG3 is segregating for a strong resistance mechanism(s) to all three ACCase herbicides.

### Selection of RG3 plants sensitive and resistant to ACCase herbicides

Of the 128 RG3 plants tested, 92 survived the ACCase herbicide treatments. The two tiller replicates for each plant and herbicide treatments behaved in a very similar way demonstrating the robustness of the tiller method for investigating the comparative efficacies of ACCase herbicides on ryegrass plants. The performance of the three FOP, DIM and DEN herbicides was highly correlated on a single plant basis indicating that a similar genetic factor may be involved in conferring resistance to all three herbicides. As cycloxydim is not known to be significantly affected by non target site resistance in ryegrass species, a target site mutation was suspected to be associated with resistance in RG3. As expected all 20 standard sensitive plants tillered and treated in the same manner as RG3 were killed with all three herbicides.

### ACCase gene analysis on sensitive and resistant RG3 and STD1 plants

PCR amplification and sequencing of eight plants each from the two sensitive and resistant RG3 subpopulations and the standard sensitive population STD1 generated an expected gene fragment of 2274 bp, showing an overall 96% homology with published ACCase ryegrass sequences. Twenty three nucleotide changes were observed among the 24 plants sequenced. Nine non-synonymous changes were detected, resulting in E1773Q, R1870P, E1874A, H1878N, E1946N, W1948R, L2012M, T2054I and C2088R mutations. Of these nine mutations, eight were present among both sensitive and resistant plants. On the other hand the cysteine to arginine mutation at codon position 2088 was present in the eight resistant RG3 plants and absent in the eight sensitive plants each from RG3 and STD1. The cysteine to arginine change results from a thymine to cytosine transition at nucleotide position 6262 of the *ACCase* gene (*Alopecurus myosuroides* equivalent). Among the eight resistant RG3 plants, five were heterozygous CR2088 and three homozygous RR2088. The combined whole plant herbicide test and sequencing results suggested that the C2088R ACCase mutation conferred dominant and strong resistance to practical field rates of clodinafop-propargyl, cycloxydim and pinoxaden in RG3.

### CAPS assay for large scale population genotyping and for confirming the association between resistance and the C2088R ACCase mutation

**Figure 1:**
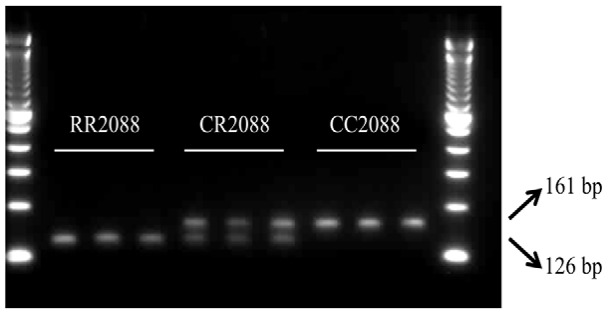
CAPS procedure for the detection of a thymine to cysteine change (C2088R mutation) at nucleotide position 6262 in *Lolium* spp. The *Hha* I digested fragment (126 bp) correspond to the resistant R2088 allele and the *Hha* 1 undigested fragment (161 bp) correspond to the C2088 allele. Heterozygous plants display both the 126 bp and 161 resistant and sensitive alleles respectively. Lanes 1 and 10: DNA ladder, lanes 2, 3 and 4: homozygous mutant RR2088, lanes 4, 5, 6: heterozygous CR2088 plants, lanes 7, 8, 9: homozygous wild CC2088 plants.

A DNA based CAPS assay was developed for genotyping a large number of RG3 plants and ascertaining the association of the C2088R mutation with clodinafop-propargyl, cycloxydim and pinoxaden resistance in RG3. PCR generated a 161 bp fragment among all the 128 RG3 and 20 plants from STD1. Upon restriction with the enzyme *Hha*I, mutant RR2088 plants generated a 126 bp fragment while wild type plants showed the undigested 161 bp fragment (Figure 1). Heterozygous plants displayed a copy each of the 161 bp and 126 bp DNA fragments. The results obtained with the CAPS and sequencing assays were completely correlated for the 24 plants sequenced earlier, thus demonstrating the accuracy of the 2088 DNA assay developed here. Genotyping of the remaining 112 RG3 plants from 128 individuals that were tillered and treated with clodinafop-propargyl, cycloxydim and pinoxaden revealed overall genotypic frequencies of 27.6% 46.9% and 25.5% for homozygous wild type CC2088, heterozygous CR2088 and homozygous mutant RR2088 plants respectively. Importantly genotype and phenotype comparison confirmed the complete association between one or two copies of the R2088 mutant allele and resistance to all three herbicides in population RG3.

The CAPS primer used here amplified successfully a corresponding ACCase gene fragment from eight plants each of the three *A. myosuroides* and *A. fatua* populations. As expected the DNA fragments were not digested upon treatment with the enzyme *Hha*I for the wild type plants from *A. myosuroides* and *A. fatua* and a clearly restricted 126 bp restricted fragment was visible on agarose gel for the resistant *A. fatua* characterised by the C2088R mutation.

### Production of pure CC2088 and RR2088 seeds and whole dose response assays with ten commonly used ACCase herbicides

Of the 387 untreated RG3 plants genotyped with the CAPS assay prior to seed bulking, 106 and 100 plants were homozygous CC2088 and homozygous RR2088 respectively. The frequencies of the CC2088, CR2088 and RR2088 were consistent with the earlier characterisation of the 128 plants treated with three ACCase herbicides. Separate crosses of 100 each of CC2088 and RR2088 plants produced similar amounts of wild and mutant seeds under optimal glasshouse conditions. Analysis of 32 individual germinated plants from each of the two lots confirmed purity of the CC2088 and RR2088 seed batches.

**Figure 2:**
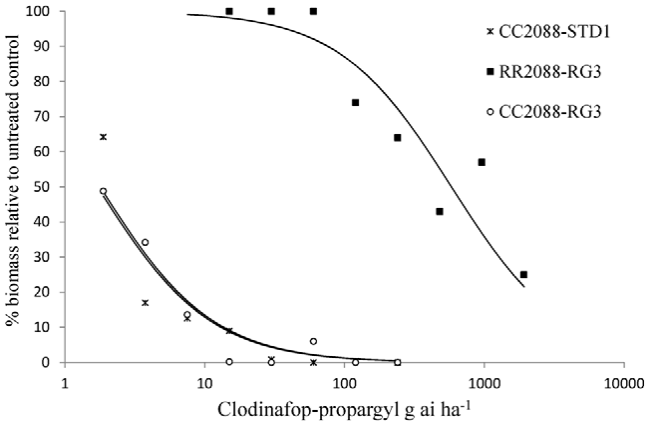
Clodinafop-propargyl whole plant dose response assay on wild and mutant 2088 genotypes.

**Figure 3:**
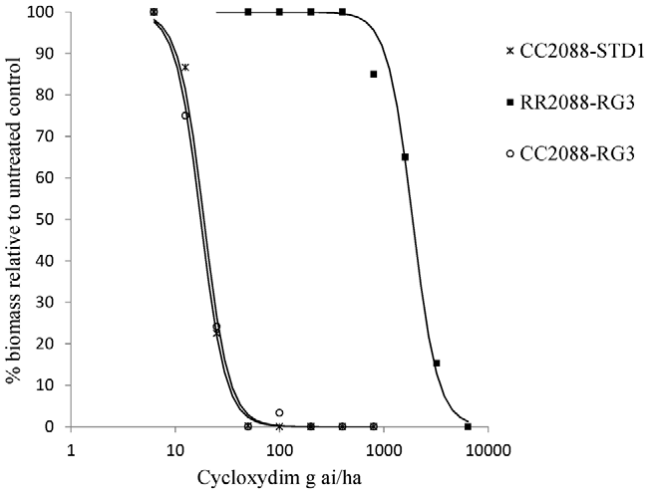
Cycloxydim whole plant dose response assay on wild and mutant 2088 genotypes.

**Figure 4:**
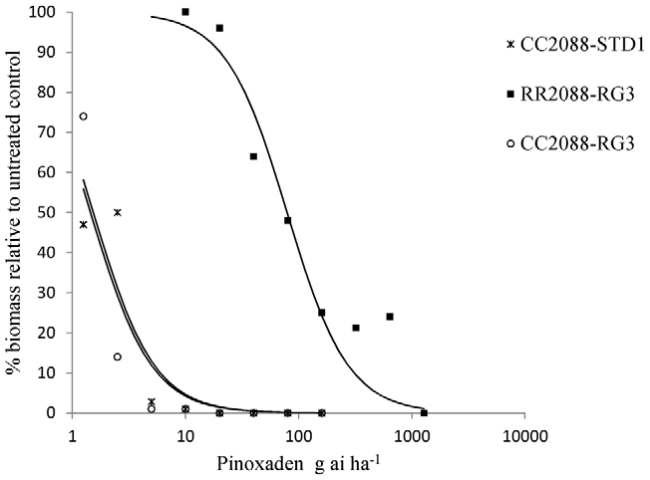
Pinoxaden whole plant dose response assay on wild and mutant 2088 genotypes.

Using split rates of herbicides for wild and mutant 2088 plants (Table 1), GR50 values could be estimated for all but mutant genotype RR2088 treated with diclofop-methyl. At the highest rate of 32000 g ai ha^−1^ diclofop-methyl, the percentage biomass reduction was 23% only. Overall, the ACCase herbicides were very active on the standard sensitive CC2088-STD1 population and the wild type CC2088-RG3 subpopulation with GR50 values ranging from less than 2 g ai ha for pinoxaden to 105 g ai ha^−1^ for diclofop-methyl (Table 2). With the exception of fluazifop (requiring the full recommended rate of herbicide), the wild type plants were controlled with half or even below, the recommended field rates of herbicides. In contrast none of the ten herbicides fully controlled the mutant RR2088-RG3 subpopulation at the common use rates and beyond for most herbicides. Corresponding resistance indices ranged from 13 and 15 for clethodim and tepraloxydim to 317 and over 358 for clodinafop-propargyl and diclofop-methyl respectively (Table 3). Interestingly, the estimated resistance factors between wild type genotypes CC2088-STD1 and CC2088-RG3 were close to 1 for all compounds, thus implying that RG3 is not characterised by additional ACCase resistance mechanisms even at very low rates of herbicides. Examples of whole plant dose responses obtained for a FOP, DIM and DEN herbicides are shown on figures 2, 3 and 4 respectively. It is noteworthy that for the most potent herbicide clethodim, the percentage biomass reduction of mutant RR2088 plants at the two-leaf stage was 55%, 85% and 90% for the commonly use rates of 60, 120 and 240 g ai ha^−1^. Though significant biomass reduction was achieved with clethodim at 120 and 240 g ai ha^−1^, several plants survived the treatments and would produce seeds.

**Table 1:** Herbicide rates used in whole plant dose response assays.

Herbicide	Rate in g ai ha^−1^ in dose response assays	
	CC2088-STD1; CC2088-RG3	RR2088-RG3
Clodinafop-propargyl	1.88, 3.75, 7.5, 15, 30, 60, 120, 240	15, 30, 60, 120, 240, 480, 960, 1920
Diclofop-methyl	31.25, 62.5, 125, 250, 500, 1000, 2000, 4000	250, 500, 1000, 2000, 4000, 8000, 16000, 32000
Fluazifop	2.11, 4.22, 8.44, 16.88, 33.75, 67.5, 125, 250, 500	8.44, 16.88, 33.75, 67.5, 125, 250, 500, 1000, 2000
Haloxyfop	0.58, 1.15, 2.34, 4.69, 9.38, 18.75, 37.5, 75	9.38, 18.75, 37.5, 75, 150, 300, 600, 1200
Quizalofop	1.17, 2.34, 4.68, 9.37, 18.75, 37.5, 75, 150, 300	4.68, 9.37, 18.75, 37.5, 75, 150, 300, 600, 1200
Cycloxydim	6.25, 12.5, 25, 50, 100, 200, 400, 800	50, 100, 200, 400, 800, 1600, 3200, 6400
Sethoxydim	9.38, 18.75, 37.5, 75, 150, 300, 600, 1200	75, 150, 300, 600, 1200, 2400, 4800, 9600
Clethodim	1.88, 3.75, 7.5, 15, 30, 60, 120, 240	15, 30, 60, 120, 240, 480, 960, 1920
Tepraloxydim	0.19, 0.39, 0.78, 1.56, 3.13, 6.25, 12.5, 25, 50	1.56, 3.13, 6.25, 12.5, 25, 50, 100, 200, 400
Pinoxaden	1.25, 2.5, 5, 10, 20, 40, 80, 160	10, 20, 40, 80, 160, 320, 640, 1280

CC2088-STD1: wild type homozygous 2088 genotype from standard sensitive population.

CC2088-RG3: wild type homozygous 2088 genotype from population RG3.

RR2088-RG3: mutant homozygous 2088 genotype from population RG3.

**Table 2:** Estimated GR_50_ values for 10 ACCase inhibiting herbicides and different 2088 ACCase genotypes.

Herbicide	CC2088-STD1	CC2088-RG3	RR2088-RG3
Clodinafop-propargyl	2.28 (1.67–3.11)	1.96 (1.08–3.53)	620.69 (358.93–1073.36)
Diclofop-methyl	105.39 (83.57–132.90)	89.34 (63.91–124.91)	>32000
Fluazifop	27.14 (25.05–29.41)	23.48 (21.09–26.13)	505.30 (430.30–592.61)
Haloxyfop	3.92 (3.50–4.40)	3.64 (3.21–4.11)	75.89 (65.65–87.72)
Quizalofop	5.07 (4.67–5.50)	5.70 (5.32–6.10)	228.17 (230.30–256.08)
Cycloxydim	18.98 (16.93–21.27)	17.57(15.58–19.81)	1873.19 (1650.73–2125.63)
Sethoxydim	23.02 (21.31–24.86)	19.76 (17.83–21.89)	2046.16 (1845.68–2268.41)
Clethodim	4.29 (4.10–4.48)	4.33 (4.18–4.49)	57.93 (55.62–60.34)
Tepraloxydim	3.43 (3.09–3.82)	3.45 (3.05–3.89)	52.00 (44.75–60.43)
Pinoxaden	1.47 (1.10–1.96)	1.61 (1.40–1.85)	83.55 (65.66–106.31)

**Table 3:** Estimated resistance factors for 10 ACCase herbicides and different 2088 ACCase genotypes.

Herbicide	RG3-CC2088 vs STD1-CC2088	RG3-RR2088 vs RG3-CC2088	RG3-RR2088 vs STD1-CC2088
Clodinafop-proparlgyl	0.86 (0.44–1.67)	317.27 (141.84–709.66)	271.98 (145.00–510.18)
Diclofop-methyl	0.85 (0.56–1.27)	>358	>304
Fluazifop	0.86 (0.76–0.99)	21.52 (17.77–26.08)	18.62 (15.58–22.26)
Haloxyfop	0.93 (0.78–1.10)	20.87 (17.26–25.25)	19.34 (16.07–23.26)
Quizalofop	1.12 (1.01–1.25)	40.06 (35.04–45.80)	45.01 (39.07–51.85)
Cycloxydim	0.93 (0.78–1.10)	106.64 (89.58–126.95)	98.71 (83.24–117.05)
Sethoxydim	0.86 (0.75–0.98)	103.57 (89.54––119.79)	88.89 (78.15–101.11)
Clethodim	1.01 (0.96–1.07)	13.37 (12.66–14.12)	13.52 (12.74–14.35)
Tepraloxydim	1.00 (0.85–1.18)	15.09 (12.45–18.30)	15.14 (12.60–18.21)
Pinoxaden	1.10 (0.79–1.51)	51.87 (39.26–68.54)	56.84 (39.00–82.86)

### Efficacy of clethodim on wild and mutant plants at the peri-emergence and one leaf stage

In an attempt to achieve full control of population RG3, clethodim the most potent herbicide was applied on smaller plants at the peri-emergence and one leaf stage. Lower levels of clethodim control were observed on the standard sensitive population at the peri-emergence stage compared to plants at the one leaf stage for all three herbicide rates tested (Table 4). Similarly partial weed control was achieved on the wild type CC2088-RG3 genotype at the peri-emergence stage for the lower clethodim rate of 60 g ai ha^−1^. This indicates that the efficacy of clethodim was suboptimal on smaller plants, especially at the peri-emergence stage, given that clethodim rates as low as 30 g ai ha^−1^ provided full control of the two wild type genotypes CC2088-STD1 and CC2088-RG3 at the two leaf stage in the previous test. In any case clethodim provided less than 40% biomass reduction on mutant plants at the peri-emergence and one leaf stages even at the highest recommended field rate of 240 g ai/ha. Pairwise comparisons using the non parametric Wilcoxon rank sum test indicated a significant difference in herbicide responses between the wild type and mutant genotypes for all but one (due to sub-optimal conditions) herbicide rate and plant growth stages, thus confirming the association between the R2088 ACCase allele and resistance to clethodim in RG3 (Table 4).

**Table 4:** Efficacy of clethodim on different 2088 ACCase genotypes and plant growth stages. Genotypes compared using Wilcoxon rank sum test.

Clethodim rate g ai ha^−1^	Plant growth Stage	Median herbicide response per genotype (% of visual control)			P-values		
		CC2088-STD1	CC2088-RG3	RR2088-RG3	CC2088-RG3 vs CC2088-STD1	RR2088 RG3 vs CC2088-STD1	RR2088-RG3 vs CC2088-RG3
60	peri-em	30	0	0	0.028	0.002	0.455
	1_leaf	45	65	35	0.054	0.34	0.009
							
120	peri-em	55	100	5	0.251	0.017	0.015
	1_leaf	70	100	40	0.123	0.162	0.013
							
240	peri-em	90	100	30	1	0.002	0.028
	1_leaf	100	100	25	0.848	0.058	0.032

## Discussion

The evolution of resistance to ACCase herbicides is seriously limiting grass weed management options in small grain cereal and dicot crops. The main objectives of this study were to conduct an integrated biological and molecular study on the mechanism of resistance to ACCase inhibiting herbicides in a *Lolium multiflorum* population and to develop a DNA based marker method for the quick identification of the target site mutation identified here.

### Mechanism of resistance to ACCase herbicides in population RG3

The study revealed a cysteine to arginine mutation in the target enzyme associated with resistance to all herbicides tested. Increasing levels of resistance were detected from clethodim to diclofop-methyl through fluazifop and pinoxaden. This is the first report of the C2088R mutation in a *Lolium multiflorum* population from the UK. The C2088R mutation has thus far been detected in five other ryegrass populations from Australia [11] and one ryegrass population from Italy [10]. The mutation was also detected in an *A. fatua* population from the USA [29]. However, in the previous studies the potential for non target site mechanisms to contribute to resistance were overlooked as the resistant populations were compared to sensitive populations from different genetic backgrounds. Here, comparison of wild and mutant RG3 subpopulations has clearly and unambiguously established the importance of the C2088R in conferring resistance to all the ten herbicides tested at the whole plant level. In addition, contrasting wild type CC2088-RG3 and CC2088-STD1 genotypes indicated that RG3 is not characterised by non target site resistance, even at very low doses of ACCase herbicides. This is a rather unique case since when analysed in detail target site resistant populations have always been found to contain underlying additional non target site resistance [9], [10], [30], [31]. For instance, in the Australian *Lolium rigidum* population SLR31, an I1781L target site resistance mutation was identified in addition to metabolic resistance [31], [32]. Subsequent genetics studies have shown that P450 mediated metabolism in this population is governed by at least two genes [22]. Similarly the *Lolium multiflorum* population UK24 was found to contain very high levels of non target site resistance to clodinafop-propargyl and diclofop-methyl in addition to the D2078G ACCase target site mutation [9]. Additional non target site resistance in populations for which a target site mutation have been uncovered could also be inferred from large number of *Lolium* spp. samples from Greece [33], USA [34], UK [35] and Italy [10] on the basis of differential responses to metabolisable and non-metabolisable ACCase herbicides. Weed populations containing additional non target site resistance mechanisms are not restricted to grass weeds as these have recently been identified in broad leaved weed populations characterised by known ALS mutations [36].

### Dynamics of target site and non target site resistance evolution under herbicide selection pressure

The presence of non target site resistance to ACCase herbicides in grass weed populations is often explained by multiple genes that individually confer low levels of resistance to herbicides, but when accumulated results in resistance to practical field rates of herbicides [23], [25]. This was demonstrated by recurrent selection at low doses of diclofop-methyl of increasingly tolerant *Lolium rigidum* plants from an initially sensitive population. Starting from a genetic pool of a few hundred plants in all, resistance to practical field rates of diclofop-methyl was acquired after three generations only. [23]. A similar scenario is believed to operate under field conditions given that not all plants receive effective rates of herbicides due to staggered germination and shading under high plant densities. Under field conditions the accumulation of minor non target site resistance genes ultimately results in significant levels of resistance to ACCase herbicides [25]. In contrast target site resistance mutations are often viewed as a rare event and present at different frequencies depending on the herbicide target [37]. Here we demonstrate that target site resistance can be selected before any non target resistance gene appears in a *Lolium multiflorum* population following years of selective ACCase herbicide selection pressure. Our observation therefore questions the idea of a large number of genes acting additively to confer non target site resistance in weeds or that ACCase target site mutations exist a low frequencies in *Lolium* spp. populations.

### Insights into herbicide binding at the ACCase target site

Crystallographic studies have recently showed that the binding mode of pinoxaden and tepraloxydim were very similar in spite of their very different chemical structures [20], [21]. Haloxyfop and diclofop on the other hand were found to share a similar binding point at a rather different region on the ACCase target [19]. Our study implies that subtle differences in binding modes of the different herbicides can have a profound effect on their efficacies in the presence of specific mutations. This can be inferred from the large differences in resistance indices between herbicides belonging to the same chemical or binding groups. Though belonging to the FOP family and found to bind very closely on ACCase, the resistance factors associated with the C2088R mutation were 20 and over 350 fold for haloxyfop and diclofop-methyl respectively. Similarly pinoxaden and tepraloxydim, identified as sharing a very close binding mode on ACCase [21], were characterised with resistance indices of 15 and 51 respectively. The differences observed between herbicide efficacies were not due to their relative potencies on *Lolium multiflorum* but due to their binding modes. This is because the resistance indices were derived based on comparison between sensitive and resistant sub-populations sharing similar genetic backgrounds except for the 2088 ACCase mutation.

### Importance of cysteine for herbicide binding and catalytic activity of ACCase

At ACCase position 2088 three different amino acids have thus far been identified. Two are sensitive to ACCase herbicides, including cysteine in *Lolium* spp. and most other grass weed species and a phenylalanine residue that is naturally present in barley, wheat and several *Bromus* species [38]. Therefore cysteine *per se* does not appear to be critical for the catalytic activity of ACCase and the effective binding of ACCase inhibiting herbicides. While this study has clearly established the detrimental effect of the C2088R on ACCase herbicide efficacies, the impact of the arginine mutation on ACCase catalytic activity and plant fitness remains to be established. In this respect, the specific activity of the wild type ACCase enzyme was higher than for the homozygous 2088 mutant enzyme [11]. It is to be noted though that the sensitive and resistant enzymes originated from different populations and that the differences in activities could be due to population to population variations. At the whole plant level and when grown under optimal glasshouse conditions, we found no visual differences in growth characteristics between the wild and mutant CC2088-RG3 and RR2088-RG3 sub-populations. The wild and mutant genotypes established here will be very useful for studying whether the C2088R ACCase mutation is affected by a fitness penalty under field conditions.

### Efficacy of clethodim on the C2088R mutant plants

Clethodim was by far the most effective herbicide on the homozygous mutant RR2088 genotypes investigated here. At the highest recommended rate of 240 g ai ha^−1^ most plants were killed. However the few individuals that survived the clethodim treatment would re-grow and produce seeds. This is in agreement with a previous study on Australian samples for which clethodim provided insufficient levels of control on C2088R resistant plants at the commonly used rate of 60 g ai ha^−1^ [11]. On the other hand clethodim at 216 g ai ha^−1^ killed all heterozygous and homozygous 2088 mutant plants from an Italian *Lolium multiflorum* population [10]. These results should nevertheless be interpreted with caution given that herbicides tend to be very active on soft (i.e. non-weathered) plants at low densities under optimal glasshouse conditions. In this respect 50% biomass reduction was achieved on sensitive CC2088-RG3 and CC2088-STD1 genotypes with less than 5 g ai ha^−1^clethodim. This level of activity is never attained under field conditions. Also it is often recommended that herbicides be applied on weeds when they are still at an early growth stage given that metabolic resistance tends to be accentuated in larger weeds. In this study however, the activity of clethodim was lower on plants at the one leaf and especially peri-emergence stage as compared to the two leaf stages. This can be explained by the fact that clethodim is a post-emergence herbicide that works optimally on plants with adequate foliage ensuring good herbicide absorption. Therefore clethodim will very unlikely control *Lolium* spp. populations containing the 2088 mutations irrespective of the plant growth stage.

### Detection of the C2088R mutation

A DNA based CAPS assay was developed for detecting the mutant R2088 allele in *Lolium* spp. The assay proved to be very robust as sequencing and CAPS results were totally correlated. The methodology used relies on the conservation of the third guanine on the 2087 ACCase codon for the identification of the R2088 allele. The 2087 codon is a glutamic acid and is particularly conserved among plant species. Glutamic acid is coded by either the GAG or GAA nucleotide triplets. Alignment of 200 ACCase sequences available in the GenBank showed that glutamate is very conserved at the 2087 position and that the codon triplet is GAG for all but one Oryza clone (CZ119516). Thus it is very unlikely that the CAPS 2088 assay developed here will be affected by false negatives for the detection of mutant R2088 alleles in *Lolium* spp. The CAPS assay complements two other dCAPS methods aimed at detecting the wild type cysteine 2088 allele and based on the positive identification of the thymine on the first base of the codon [10], [39]. The method developed by Scarabel et al [10] also relies on the conservation of the last base of the 2087 codon for unambiguous detection of the wild type cysteine allele while the reverse primer from the Delye et al [39] assay encompass all the bases of the recognition site of the restriction enzyme used except for the variable first base of the 2088 codon. However, the latter assay uses a nested PCR strategy for successful amplification of the ACCase gene fragment, thus adding an additional and costly step in the detection procedure. The interest of the current CAPS 2088 assay is that it is highly transferable to other grass weed species namely *A. fatua* and *A. myosuroides*, prone to evolve resistance to ACCase herbicides. It employs a very efficient restriction enzyme which is two and four times cheaper than the ones used in the assays proposed by Scarabel et al [10] and Delye et al [39] respectively. It is thus particularly suited for large scale genotyping of ryegrass populations as is the case in this study. Additionally, because it is a CAPS assay, it is less likely to be affected by priming stability compared to dCAPS assays with one [10] and two [39] forced mutations in the reverse primers.

In conclusion, we identified a C2088R target site mutation conferring high levels of resistance to a broad range of ACCase herbicides in the *Lolium multiflorum* population from the UK. The population is rather unique as it does not appear to contain any additional underlying non target site resistance mechanisms affecting the efficacies of all ACCase herbicides studied here. The C2008R mutation can be detected using a robust CAPS assay that is readily transferable to two other grass weed species. Additionally the pure CC2088 and RR2088 sub-populations created as part of this study will be helpful in determining whether the R2088 allele confers a fitness penalty in competition under field conditions.

## Materials and Methods

### Plant materials

Seeds from the suspected resistant *Lolium multiflorum* population (RG3) were collected in 2003 from a wheat field in Widnes, Cheshire, UK. The seeds were randomly sampled from a large number of surviving plants from across the entire field. A standard sensitive *Lolium multiflorum* population (STD1) commercially available from Herbiseed (Twyford, Berkshire, UK) was used for comparison in all glasshouse and DNA tests. Additionally, a sensitive *Alopecurus myosuroides* population and a sensitive and resistant *Avena fatua* populations were used for testing the transferability of the DNA based Cleaved Amplified Polymorphic Sequence (CAPS) marker developed in this study to other species.

“No specific permissions were required for the location where the ryegrass seeds were collected. This study did not involve any endangered or protected species”.

### Initial resistance confirmation test

Around 50 seeds for each of STD1 and RG3 populations were sown in individual pots containing a 1∶1 ratio of compost and peat and irrigated as necessary. The germinated plants were maintained in a controlled greenhouse set at 24°C/16 hr day, 18°C night, 65% relative humidity conditions, and a photon flux density of approximately 250 μmol quanta m^–2^ s^–1^. The plants at the two leaf stage were treated with a single rate of commercial formulations of clodinafop-propargyl (60 g ha^−1^), cycloxydim (100 g ha^−1^) and pinoxaden (45 g ha^−1^). Three replicate pots were used and the level of control was visually assessed 21 days after treatment.

### Segregation of the RG3 population into plants sensitive and resistant to ACCase herbicides

Seeds were sown as described earlier. One hundred and twenty eight seedlings were transplanted in individual pots and allowed to tiller by trimming the plants regularly. Two months later the 128 mother plants were separated into seven genetically identical tillers. The roots and leaves were trimmed and the tillers from each mother plant were potted separately. After one week, two separate tillers each from the 128 mother plants were treated with clodinafop-propargyl (60 g ha^−1^), cycloxydim (100 g ha^−1^) and pinoxaden (45 g ha^−1^), whilst the 7^th^ tiller was used as untreated control. Twenty plants from the standard sensitive *Lolium multiflorum* population STD1 were also tillered and sprayed in the same way as the resistant population. Twenty one days after treatment the 128 mother plants were classified as either dead and sensitive or alive and resistant to the ACCase herbicides.

### Sequencing of the carboxyl transferase (CT) domain of the ACCase gene

A gene fragment comprising the entire carboxyl transferase domain of *ACCase* was sequenced using an RT-PCR procedure. Eight plants each of sensitive and resistant RG3 subpopulations and eight other plants from the standard sensitive population STD1 were sequenced. For each of the 24 plants, 1 g of frozen leaf tissue was ground using a pestle and a mortar in the presence of liquid nitrogen and extracted using the Trizol procedure (Invitrogen, CA, USA). One µg of RNA from each plant was cleared from contaminating DNA using a DNase treatment (Invitrogen, CA, USA). First strand DNA syntheses were carried out using purified RNA and Oligo-dT and reverse transcriptase superscript II (Invitrogen, CA, USA). Gene specific primers were used for the second DNA strand synthesis, and were based on available Genbank *Lolium* spp. ACCase sequences (AF359513, AF359514, AF359515 and AF359515) for the forward primer (5′TGGTCCTTTGCATGGTGTCGC3′) and consensus reverse primer (5′GCTCTCTTAGAGGGATCCATCTTATC3′) deduced following alignment of *Setaria italica* (AF294805), *Alopecurus myosuroides* (AJ310767), and *Zea mays* (ZMU19183) ACCase sequences. The final PCRs for second strand DNA synthesis were carried out using puReTaq Ready-To-Go PCR beads (Amersham Biosciences, Bucks, UK) in a total volume of 25 µL containing 0.8 µM of each primer and 1 µL product from 1^st^ strand DNA synthesis. PCRs were performed on an Eppendorf Master Cycle Gradient Thermocycler Model 96 programmed for an initial denaturation step of 94°C of 2 min followed by 40 cycles of 30 s at 94°C, 30 s at 64°C and 2 min at 72°C. A final extension step for 10 min at 72°C was also included. ACCase sequence alignment and comparison was carried out with the Seqman software (Lasergene, USA).

### Development of a Cleaved Amplified Polymorphic Sequence (CAPS) method for detecting the C2088R ACCase mutation

A DNA based Cleaved Amplified Polymorphic Sequence (CAPS) procedure was developed for large scale genotyping of the 128 individuals treated with clodinafop-propargyl, cycloxydim and pinoxaden and for confirming the association of the C2088R mutation with resistance to ACCase herbicides. The wild and mutant amino acid residues at ACCase position 2088 are coded by the triplets TGC and CGC respectively. The discriminant enzyme employed in the CAPS assay was *Hha*I. This enzyme recognises the quartet GCGC (arginine) from mutant 2088 genotypes but not the corresponding GTGC (cysteine) sequence from the wild type plants. As cysteine can be potentially coded by the triplets TGC or TGT and the second base of the 2088 codon is always a G in *Lolium* spp., the 2^nd^ and 3^rd^ bases of the 2088 codon was enforced with the last two bases at the 3′ end of the reverse CAPS primer. For each plant, a 2 cm leaf segment was ground on a Spex Certiprep (Metuchen, NJ, USA) 2000 model Genogrinder. DNA from the ground material was subsequently extracted on a Biomek FX robot (Beckman Coulter, High Wycombe, Buckinghamshire, UK) using a Wizard Magnetic 96 DNA Plant System kit (Promega, Madison, WI, USA).

PCR amplification was carried out with the forward 5′TCAACAATTGTTGAGAACCTTAGG3′ and reverse 5′AGAACATTCCCTTTTGCAGTTGTCTCAGCATAGC3′ primers to generate a 161 bp PCR fragment. PCRs were carried out using puReTaq Ready-To-Go PCR beads (Amersham Biosciences, Bucks, UK) in a total volume of 25 µL containing 0.8 µM of each primer and from 10–50 ng genomic DNA. PCRs were performed on an Eppendorf Master Cycle Gradient Thermocycler Model 96 programmed for an initial denaturation step of 94°C of 2 min followed by 40 cycles of 30 s at 94°C, 30 s at 64°C and 1 min at 72°C. A final extension step for 10 min at 72°C was also included.

Two µl aliquots of neat PCR products were digested with 2 units of *Hha*I (New England Biolabs, Hertfordshire, UK) in a total volume of 20 µL according to the manufacturer's recommendations and analysed on 2% agarose/1 X TBE buffer gels containing 0.5 µg mL^−1^ ethidium bromide. Upon restriction with *Hha*I the mutated allele is digested into a 126 bp and 35 bp (invisible on the 2% agarose gel) fragments.

To test the transferability of the CAPS method to two other major grass weed species affected by ACCase herbicide resistance, eight plants each from a sensitive *A. myosuroides*, a sensitive *A. fatua* and a resistant *A. fatua* populations characterised by the C2088R mutation were extracted and analysed as described for the ryegrass populations.

### Production of pure homozygous CC2088 and RR2088 seeds and whole plant dose response tests with 10 different ACCase herbicides

Three hundred and eighty seven RG3 plants were grown and genotyped with the DNA based CAPS method as described above. One hundred plants each of the CC2088 and RR2088 genotypes were separated and allowed to cross freely to produce pure wild type and mutant 2088 seeds. Subsequently 32 seeds from each of the two batches were germinated and tested for purity using the 2088 CAPS marker. These pure wild and mutant seed lots were subsequently used in whole plant dose response assays for assessing the precise resistance indices (RI) associated with the C2088R mutation. The standard sensitive population STD1 was tested alongside to determine whether other additional underlying mechanisms contribute to resistance to the ACCase herbicides. The ACCase herbicides and the rates used for each genotype in dose response assays are given in table 1. For each herbicide rate, four replicates pots containing six plants at the two leaf stage each were tested. Visual biomass relative to untreated controls was assessed 21 days after treatment.

The resulting percentages were analysed using a non-linear least squares regression model with the objective of estimating the resistance factors between the different genotypes for each herbicide. Separate regression lines were fitted to the data for each genotype but with the slope of each line constrained to be identical for all genotypes. For each genotype, the fitted model is sigmoidal in shape and described by the equation:
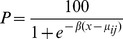
where x denotes log_10_(Rate), µ_ij_ denotes the logGR50 for genotype i and β denotes the common slope fitted to all regression lines. The estimated resistance index between a pair of genotypes is calculated as the ratio of their estimated GR50 values. Confidence intervals around the estimated resistance indices were calculated using the error term derived from a separate analysis of variance having excluded from the analysis those treatments that did not contribute meaningfully to the estimated residual sum of squares. All statistical analyses were carried out using SAS software, version 9.2.

### Influence of plant growth stage on clethodim efficacy

To investigate whether effective weed control could be achieved on smaller RG3 plants, wild and mutant 2088 genotypes were treated at the peri-emergence (around the time of emergence) and one leaf stage with clethodim, the most potent herbicide on 2088 mutant plants as identified with the whole plant dose response assays. Six replicates pots of ten plants each of the standard sensitive CC2088-STD1, wild type CC2088-RG3 and mutant type RR2088-RG3 genotypes were grown at the appropriate growth stages and tested at 60, 120 and 240 g ha^−1^ of clethodim. The pots were arranged according to a randomised complete block (RCB) design. Visual biomass relative to untreated controls was assessed 21 days after treatment. The data for each treatment and genotype were analysed using the nonparametric Wilcoxon rank sum test. Pairwise comparisons were carried out between two genotypes for each herbicide rate and plant growth stage. A p-value of less than 0.05 indicates a statistically significant result at the 5% probability level and therefore provides evidence that the median levels of control produced by the two genotypes in question are genuinely different.
